# Correction: Knowledge, attitude, and perceptions towards the 2019 Coronavirus Pandemic: A bi-national survey in Africa

**DOI:** 10.1371/journal.pone.0247351

**Published:** 2021-02-17

**Authors:** Hager Elnadi, Ismail A. Odetokun, Obasanjo Bolarinwa, Zainab Ahmed, Ochulor Okechukwu, Ahmad I. Al-Mustapha

The first and last name of the first author are incorrectly switched. The correct name is: Hager Elnadi. The first and last name of the fourth author are also incorrectly switched. The correct name is: Zainab Ahmed. The correct citation is: Elnadi H, Odetokun IA, Bolarinwa O, Ahmed Z, Okechukwu O, Al-Mustapha AI (2020) Knowledge, attitude, and perceptions towards the 2019 Coronavirus Pandemic: A bi-national survey in Africa. PLoS ONE 15(7): e0236918. https://doi.org/10.1371/journal.pone.0236918
