# Supplementary material for: Knowledge, attitude, and perceptions towards the 2019 Coronavirus Pandemic: A bi-national survey in Africa
Source: PLoS One. 2020 Jul 29;15(7):e0236918. doi: 10.1371/journal.pone.0236918 (PMC7390376; doi:10.1371/journal.pone.0236918)
Supplement: S1 Table — (DOCX) [file pone.0236918.s001.docx]

Table s1: Descriptive statistics (Correct answer rate) of knowledge of COVID-19 pandemic in Nigeria and Egypt.

| 1. Have you heard of the COVID-19 | No. of respondents (%) |
| --- | --- |
| Maybe | 1 (0.07) |
| No | 3 (0.21) |
| Yes | 1433 (99.72) |
| Total | 1437 (100%) |
| 1. Source of Information |  |
| Friends/family | 350(24.3) |
| Internet (Social media) | 1204 (83.7) |
| Newspapers | 303 (38.2) |
| Other sources | 52 (3.6) |
| TV | 631 (43.9) |
| 1. Is COVID-19 the same as the common flu? |  |
| I don't know | 94 (6.54) |
| No | 1127 (77.87) |
| Yes | 216 (15.03) |
| Total | 1437 (100) |
| 1. Is it possible to asymptomatic COVID-19 infections? |  |
| I don't know | 32 (2.23) |
| No | 210 (14.61) |
| Yes | 1195 (83.16) |
| Total | 1437 (100) |
| 5. what is the incubation period? |  |
| 1 - 14 days | 1365 (94.99) |
| 1 - 3 months | 5 (0.35) |
| 2 - 21 days | 56 (3.9) |
| I don't know | 11 (0.77) |
| Total | 1437 (100) |
| 6. Who can get infected with COVID-19? |  |
| Anyone can be infected | 1422 (98.96) |
| Older people only | 8 (0.56) |
| People with chronic diseases only | 6 (0.42) |
| Teenagers and children only | 1 (0.07) |
| Total | 1437 (100) |
| 7. Symptoms of COVID-19 |  |
| Bleeding | 69 (4.8) |
| Dry cough | 1334 (92.8) |
| Difficulty breathing | 1372 (95.4) |
| Fatigue | 888 (61.8) |
| Hair loss | 17 (1.1) |
| Muscle pain | 458 (31.8) |
| High fever | 1349 (93.8) |
| Runny nose | 605 (42.1) |
| 8. Mode of transmission |  |
| Air droplets (from patient sneezing/coughing) | 1361 (94.7) |
| Close contact with people who have the virus | 1301 (90.5) |
| Contact with contaminated surfaces | 1227 (85.3) |
| Mosquitos/flies bites | 5 (0.3) |
| 9. Viral inactivation |  |
| Alcohol - based sanitizers | 1330 (92.5) |
| Clean surfaces with diluted chlorine | 785 (54.6) |
| I don't know | 71 (4.9) |
| Soap/detergents | 1163 (80.9) |
| Water alone | 53 (3.6) |
| 10. Is handwash important? |  |
| Maybe | 2 (0.14) |
| No | 1 (0.07) |
| Yes | 1434 (99.79) |
| Total | 1437 (100) |
| 11. For how long should you wash your hands |  |
| > 5minutes | 73 (5.08) |
| 1 minute to 3 minutes | 112 (7.79) |
| 20 seconds to 1 minute | 1037 (72.16) |
| 3 minutes to 5 minutes | 80 (5.57) |
| I don't know | 52 (3.62) |
| Less than 20 seconds | 83 (5.78) |
| Total | 1437 (100) |
