# Supplementary material for: Knowledge, attitude, and perceptions towards the 2019 Coronavirus Pandemic: A bi-national survey in Africa
Source: PLoS One. 2020 Jul 29;15(7):e0236918. doi: 10.1371/journal.pone.0236918 (PMC7390376; doi:10.1371/journal.pone.0236918)
Supplement: S2 Table — (DOCX) [file pone.0236918.s002.docx]

Table s2 Descriptive statistics (Correct answer rate) of attitude towards preventive measures to the COVID-19 pandemic in Nigeria and Egypt.

| 1. Which is Protective against COVID-19? | No. of respondents (%) |
| --- | --- |
| Proper hygiene (handwash/cover mouth and nose during coughing or sneezing) | 1396 (97.1) |
| Self - Isolation/ Social distancing | 1129 (78.56) |
| Face masks/gloves | 1161 (80.8) |
| Garlic, Onions, and Ginger | 265 (18.4) |
| 1. Who can get infected? |  |
| Everyone | 861 (59.9) |
| People in contact with the ill | 635 (44.2) |
| Only sick people | 473 (32.9) |
| Health workers | 622 (43.3) |
| 1. Does Social distancing can help control COVID-19 |  |
| I don't know | 2 (0.14) |
| No | 22 (1.53) |
| Yes | 1381 (96.1) |
| Maybe | 32 (2.23) |
| Total | 1437 (100) |
| 1. The ideal distance between people |  |
| >5 meters | 122 (8.49) |
| 1 - 2 meters | 923 (64.23) |
| 3 - 5 meters | 338 (23.52) |
| I don't know | 32 (2.23) |
| Less than 1 meter | 22 (1.53) |
| Total | 1437 (100) |
| 1. Do you follow the COVID-19 recommendations? |  |
| No | 17 (1.18) |
| Sometimes | 91 (6.33) |
| Yes | 1329 (92.48) |
| Total | 1437 (100) |
| 1. If yes, to what extent? |  |
| I do not follow any of the recommendations | 1 (0.07) |
| I follow all the recommendations | 519 (36.12) |
| I follow most of them | 688 (47.88) |
| I follow some but not all | 221 (15.38) |
| Not at all | 8 (0.56) |
| Total | 1. (100) |
| 1. Frequency of face touching |  |
| Always | 97 (6.75) |
| Never | 62 (4.31) |
| Often | 294 (20.46) |
| Rarely | 478 (33.26) |
| Sometimes | 506 (35.21) |
| Total | 1437 (100) |
| 1. How do you feel? |  |
| Angry | 217 (15.1) |
| Bored | 749 (52.1) |
| Fear | 634 (44.1) |
| Happy | 20 (1.39) |
| Having sleep problems | 141 (9.8) |
| Just fine | 186 (12.9) |
| Lonely | 260 (18) |
| Nervous/Anxious | 681 (47.4) |
| Relaxed/optimistic | 257 (17.8) |
| Stressed | 316 (22) |
| 1. How are you adapting? |  |
| Watching TV/movies | 971 (67.57) |
| Following Social media (Facebook/WhatsApp/Instagram) | 1207 (83.99) |
| Volunteering | 184 (12.8) |
| Working from home | 502 (34.93) |
| Practicing indoor sports | 372 (25.88) |
| Reading books/magazines | 793 (50.8) |
| Playing Video Games | 167 (11.62) |
| Sleeping all the time | 166 (11.55) |
| Spending time with family | 940 (65.41) |
| Fighting with everyone around | 7 (0.49) |
| Talking to myself | 155 (10.78) |
| 1. Stress/worry rating |  |
| 1 | 181 (12.6) |
| 2 | 269 (18.72) |
| 3 | 552 (38.41) |
| 4 | 248 (17.26) |
| 5 | 187 (13.01) |
| Total | 1437 (100) |
