# Supplementary material for: Knowledge, attitude, and perceptions towards the 2019 Coronavirus Pandemic: A bi-national survey in Africa
Source: PLoS One. 2020 Jul 29;15(7):e0236918. doi: 10.1371/journal.pone.0236918 (PMC7390376; doi:10.1371/journal.pone.0236918)
Supplement: S3 Table — (DOCX) [file pone.0236918.s003.docx]

Table s3: Descriptive statistics (Correct answer rate) of perception of the global response to the COVID-19 pandemic in Nigeria and Egypt.

| 1. Do you think that your government has done enough? | No. of respondents (%) |
| --- | --- |
| I don't know | 9 (0.63) |
| Maybe | 288 (20.04) |
| No | 586 (40.78) |
| Yes | 554 (38.55) |
| Total | 1437 (100) |
| 1. Do you agree with compulsory lockdown? |  |
| Maybe | 136 (9.46) |
| No | 138 (9.6) |
| Yes | 1163 (80.93) |
| Total | 1437 (100) |
| 1. Has WHO done enough? |  |
| Maybe | 367 (25.54) |
| No | 365 (25.4) |
| Yes | 705 (49.06) |
| Total | 1437 (100) |
| 1. Your countries response to the pandemic |  |
| 1 | 246 (17.12) |
| 2 | 341 (23.73) |
| 3 | 523 (36.4) |
| 4 | 226 (15.73) |
| 5 | 101 (7.03) |
| Total | 1437 (100) |
| 1. Rating of the social media coverage of the COVID-19 pandemic |  |
| Very satisfied/keeps me updated | 702 (48.85) |
| Makes me worry/more stressed | 207 (14.4) |
| Not enough information | 194 (13.5) |
| There are more lies than truth | 308 (21.4) |
| I don't follow any media updates | 33 (2.29) |
| No comments | 99 (6.89) |
