# Supplementary material for: Knowledge, attitude, and perceptions towards the 2019 Coronavirus Pandemic: A bi-national survey in Africa
Source: PLoS One. 2020 Jul 29;15(7):e0236918. doi: 10.1371/journal.pone.0236918 (PMC7390376; doi:10.1371/journal.pone.0236918)
Supplement: S4 Table — (DOCX) [file pone.0236918.s004.docx]

Table s4: Descriptive statistics (Correct answer rate) of respondents to community response associated with the prevention of a future pandemic.

| 1. What can a community do to reduce the spread? | No. of respondents (%) |
| --- | --- |
| Follow/respect the Health recommendations of my country | 1340 (93.24) |
| Eat Healthy/ Practice sports | 823 (57.27) |
| Social distancing/Avoid the crowd | 1277 (88.86) |
| Volunteer to support whenever possible | 731 (50.86) |
| Avoid handshakes and face kissing | 1170 (81.41) |
| Attending religious gatherings | 21 (1.46) |
| I don't know | 7 (0.49) |
| 1. Can we prevent future pandemics? |  |
| I don't know | 22 (1.53) |
| Maybe | 259 (18.02) |
| No | 211 (14.68) |
| Yes | 945 (65.76) |
| Total | 1437 (100) |
| 1. Preventing future global pandemic |  |
| Reduce international travels | 472 (32.8) |
| Establish early alerts and global warning systems for infectious diseases | 1175 (81.8) |
| Collaboration between environmental, animal and human health workers | 912 (63.5) |
| Intensify research on preventive measures such as vaccines/diagnosis | 1156 (80.4) |
| Improve surveillance in the human and animal health sectors | 1044 (72.65) |
| Raise public awareness of proper hygiene/healthy habits | 1030 (71.6) |
| Prioritize human life/health welfare over the animal or environmental ones | 340 (23.6) |
| 1. Willingness to share info |  |
| Maybe | 86 (5.98) |
| No | 49 (3.41) |
| Yes | 1302 (90.61) |
| Total | 1437 (100) |
